# B-Lynch Suture Management among Patients with Postpartum Hemorrhage in a Tertiary Care Centre: A Descriptive Cross-sectional Study

**DOI:** 10.31729/jnma.8036

**Published:** 2023-02-28

**Authors:** Poonam Koirala, Asmita Ghimire, Kesang Diki Bista

**Affiliations:** 1 Department of Obstetrics and Gynaecology, Tribhuvan University Teaching Hospital, Maharajgunj, Kathmandu, Nepal

**Keywords:** *cesarean section*, *postpartum haemorrhage*, *suture*

## Abstract

**Introduction:**

Postpartum haemorrhage is the most life-threatening complication during pregnancy and atonic postpartum haemorrhage being the commonest one, often poses difficulties in management. B-Lynch suture with a high success rate has emerged as a life-saving measure in uncontrolled atonic postpartum haemorrhage refractory to uterotonics. The objective of this study was to find out the prevalence of B-Lynch suture management among patients with post-partum haemorrhage in a tertiary care centre.

**Methods:**

This descriptive cross-sectional study was conducted in the Department of Obstetrics and Gynaecology of a tertiary care centre from 1 April 2017 to 1 April 2021 after taking ethical approval from the Institutional Review Committee of the same institution [Reference number: 497(6-11) C-2077/078]. All patients with post-partum haemorrhage during the study period were included in the study. Patients with traumatic post-partum haemorrhage, congenital malformations, complete placenta previa/accreta, bleeding disorders, disseminated intravascular coagulation, and retained bits of placenta were excluded from the study. A convenience sampling method was used. Point estimate and 90% Confidence Interval were calculated.

**Results:**

Out of 72 patients, 19 (26.39%) (17.85-34.93, 90% Confidence Interval) underwent B-Lynch suture management for atonic post-partum haemorrhage. Uterus salvage was done in 18 (94.74%) whereas 1 (5.26%) underwent a cesarean hysterectomy.

**Conclusions:**

The prevalence of the use of B-Lynch suture was similar to other studies done in similar settings. B-Lynch suture is a valuable addition for controlling intractable atonic primary postpartum haemorrhage refractory to uterotonics, thus saving the life as well as preserving the future fertility of the woman.

## INTRODUCTION

Post-partum haemorrhage (PPH) is a leading cause of global maternal mortality and morbidity, accounting for 25-30% of all maternal deaths, and 75-90% of these casualties result from uterine atony.^12^ It may lead to a cesarean hysterectomy thus impairing future fertility. Uterine atony accounts for more than 80% of cases of primary PPH. PPH may occur after vaginal delivery 4% or cesarean births 6%.^3,4^

B-Lynch provides compression to both sides of the uterine body without disturbing the anatomy. It can stop postpartum haemorrhage without the need for pelvic surgery and potentially preserve fertility.^5^ The success rate of B-Lynch in avoiding hysterectomy is 86.4% and has been widely recommended for controlling PPH.^6^ Thus, the practice of this surgical management in the country is following the rising curve but there is a dearth of published literature regarding this topic.

The aim of this study was to find out the prevalence of B-Lynch sutures management among patients with post-partum haemorrhage in a tertiary care centre.

## METHODS

This descriptive cross-sectional study was conducted in the Department of Obstetrics and Gynaecology of Tribhuvan University Teaching Hospital from 1 April 2017 to 1 April 2021. The study was conducted after taking ethical approval from the Institutional Review Committee of the Institute of Medicine [Reference number: 497(6-11 )C-2077/078]. All the patients who were delivered in the hospital and who had atonic PPH during the study period were included in this study. Patients with traumatic PPH, congenital malformations, complete placenta previa/accreta, bleeding disorders, disseminated intravascular coagulation (DIC), and retained bits of placenta were excluded from the study. Convenience sampling was used. The sample size was calculated using the following formula:


$$\begin{array}{ll} \text{n} & ={\text{Z}}^{2}\times \frac{\text{p}\times \text{q}}{{\text{e}}^{\text{2}}}\\ & =1.64^{2}\times \frac{0.5\times 0.5}{0.10^{2}}\\ & =68 \end{array}$$

Where,

n = minimum required sample size
Z = 1.645 at 90% Confidence Interval (CI)
p = prevalence taken as 50% for maximum sample size calculation
q = 1-p
e = margin of error, 10%

The calculated sample size was 68. However, 72 samples were taken in this study.

The active management of the third stage of labour was done in all the cases (controlled cord traction, uterine massage, and oxytocin). Despite using uterotonics in atonic PPH if the patients had intractable haemorrhage then a B-Lynch brace suture was applied. In this study, the patients with the primary PPH who didn't respond to uterotonics and managed with B-Lynch suture application due to uncontrollable haemorrhage were included.

Data were collected from the labour room confinement book and cesarean section record book. The proforma for data collection included parity, mode of delivery, an indication of cesarean delivery, blood transfusion, additional surgical procedures, and need for Intensive Care Unit (ICU). After managing atonic PPH with medical drugs B-Lynch suture was applied with Polydioxanone Vicryl 01 suture. The effectivity was simply judged by the stoppage of bleeding after B-Lynch suture application. Any complications during the first five days were recorded from the patient file which included further treatment if bleeding re-occurs, postoperative fever, ICU/Critical Care Unit (CCU) / Ventilator support, DIC, hysterectomy, and maternal death.

The collected data were entered and analyzed using IBM SPSS Statistics version 22.0. Point estimate and 90% CI were calculated.

## RESULTS

Among 72 patients with post-partum haemorrhage, 19 (26.39%) (17.85-34.93, 90% CI) underwent B-Lynch suture management. Among the patients with B-Lynch application, in 1 (5.26%) women during the cesarean section for short spacing, the uterus became flabby and did not respond to uterotonics and uterine massage. B-Lynch suture was applied following which bleeding was reduced. After 6 hours again patient had an intractable haemorrhage, and ultimately cesarean hysterectomy was performed to save her life (Table 1).

**Table 1:** Post-operative findings in patients who had B-Lynch application (n= 19).

Post-operative findings	n (%)
Uterus salvage: bleeding stopped/ reduced after application	18 (94.74)
Cesarean hysterectomy: bleeding persisted even after application	1 (5.26)

There was 1 (5.26%) maternal mortality in heart disease patient with pulmonary stenosis with severe mitral regurgitation 11 hours later due to cardiac arrest. However, the bleeding stopped after the B-Lynch suture application. A total of 3 (15.79%) patients developed wound infection and 15 (78.95%) were discharged without complications. None of the patients reported to the hospital for any complications. The mean age of patients was 28.5 (Range: 19-39) years. The mean birth weight of the baby was 3.42 kg with 12 (60%) babies having birth weight >3.0 kg. A total of 11 (57.98%) were primipara and most of the patients 8 (42.10%) were of the 26-30 years age group (Table 2).

**Table 2:** Demographic and clinical profile of patients with B-Lynch suture application (n= 19).

Characteristics	n (%)
**Age (years)**	
19-25	1 (5.26)
26-30	8 (42.10)
31-35	6(31.57)
>35	4(21.05)
**Parity**	
Primipara	11 (57.89)
≥P2	8 (42.10)
**Gestational age (weeks)**	
<32	-
32-37	7 (36.84)
37-40	10 (52.63)
40-42	2 (10.52)
Birth weight (kg)	(n= 20)^*^
<2	2 (10)
2-2.9	6 (30)
3-3.9	9 (45)
≥4	3 (15)

* One twin delivery

A total of 14 (73.68%) women had an emergency cesarean delivery. Fetal distress seen in 6 (31.58%) was the commonest indication of the cesarean section followed by previous cesarean section in 4 (21.05%) (Table 3).

**Table 3:** Caesarean section details among patients with B-Lynch application (n= 19).

Parameters	n (%)
**Type of caesarean section**	
Emergency	14 (73.68)
Elective	5 (26.31)
**Indications of caesarean section**	
Fetal distress	6 (31.57)
Previous cesarean section	4 (21.05)
Abruption placenta with the low-lying placenta	1 (5.26)
Pronged premature rupture of membranes (PROM)	1 (5.26)
Big baby >4 kg	1 (5.26)
Multiple pregnancies	1 (5.26)
Idiopathic thrombocytopenia	1 (5.26)
Eclampsia	1 (5.26)
Failed vacuum	1 (5.26)
Pulmonary stenosis	1 (5.26)
Low lying placenta	1 (5.26)

The average blood loss was 1784 ml and 13 (68.42%) patients had blood loss between 1000-2000 ml, whereas 5 (26.32%) cases had blood loss of more than 2000 ml. Blood transfusion was not required in 4 (21.05%) patients, whereas 3 (15.78%) required more than or equal to three units of blood transfusion (Table 4).

**Table 4:** Blood loss and transfusion among patients with B-Lynch application (n= 19).

Distribution	n (%)
**Blood loss (ml)**	
500-1000	2(10.52)
1000-2000	12(63.17)
2000-3000	3(15.78)
>3000	2 (10.52)
**Units of blood transfusion**	
No blood transfusion	4(21.05)
1 unit	8(42.10)
2 units	4(21.05)
>3 units	3(15.78)

## DISCUSSION

Atonic PPH has always been a challenging task for obstetricians to manage. Recently emerged B-Lynch suture has been quite valuable in treating atonic PPH refractory to uterotonics, which not only preserves future fertility but also is life-saving In our present study the prevalence of B-Lynch suture was 26.4% and 94.7% successful in controlling atonic PPH refractory to uterotonics.

PPH often leads to litigation issues if maternal death happens. Obstetricians always have to work under this fear of deadly complications which if uncontrolled can lead to worse consequences. The emergence of B-Lynch suture recently has been of great help in controlling intractable haemorrhage. It is simple and effective, leads to satisfactory hemostasis after application, and if it fails we still have other radical procedures. This technique is simple, requires less time, and can be used in emergencies to preserve fertility and life. WHO guidelines state that after the failure of conservative management, compression sutures should be attempted before vessel ligations.^7^ There are some reports where B-Lynch was used only for control of postpartum haemorrhage, without any vessel ligation and the pregnancy outcomes in these cases were favourable.^8^ Uterine compressive sutures are a well-established measure for control of haemorrhage following atonic postpartum haemorrhage when medical and nonmedical interventions fail.^9^ The absorbable suture can be left in situ, and would typically not lead to problems with future pregnancies.^10^

In the present study, the mean age of patients was quite similar to the study done in India (26.8 years),^11^ but contrary to the study conducted in Singapore (35 years).^12^ This may be due to early marriages and childbearing in our developing country. This is also similar to a study from India,^13^ in which the mean age of patients was 26.6 years. This age difference may be due to early marriages and childbearing in our society due to cultural customs as per religion, socioeconomic status of the population, and country. The mean gestational age was 37.8 weeks (32-41). The results are similar to the following studies.^11-14,15^

Atonic PPH was most common in primipara, whereas in a study conducted in Mumbai, India, atonicity was equal in both primipara and multiparous though the difference is not much in the present study too.^13^ This is in contrast to findings in a study from India where the majority of patients were multigravida.^16^ This can be attributed to different causative factors in different populations and indications of cesarean section in such women.

The average birth weight in the present study is similar to a study conducted in Scotland in which the birth weight was 3.5 kg.^15^ In the present study emergency, cesarean section was the commonest mode of delivery which is quite similar to a study done in India which showed that 76% of patients had emergency LSCS and only 24% had elective LSCS.^17^ This indicates that PPH most commonly occurs in emergency LSCS. Similarly in a study from India, 76% had emergency CS and 23.52% had an elective cesarean section.^11^

Fetal distress followed by previous cesarean section was the commonest indication of cesarean section in our study, whereas a study from India showed prolonged labour (33%) followed by antepartum haemorrhage (30%) and prelabour rupture of membrane (20%) were the causative factors of atonic PPH.^13^ In another study from India Preeclampsia was the most common cause, whereas in our study fetal distress was the commonest cause of cesarean delivery resulting in atonic PPH later.^11^

The average blood loss in the present study was 1784 ml. The maximum number of patients had blood loss in the range of 1000-2000 ml. It indicates timely application and thus reduced blood transfusion, with only a few patients exceeding blood loss ≥2000 ml. The mean blood loss was higher in comparison to studies (1363 ml, 1480 ml),^13,14^ this difference may be due to difficulty in accurate assessment of blood loss. Assessment of blood loss may not be accurate by measuring the mops soaked as there is blood loss in the patient sheet and also some amount of blood is mixed with amniotic fluid during delivery. Extensive blood loss of >2000 ml was seen in one patient.^15^ In our patients, B Lynch suture was timely applied so probably due to that the amount of blood loss and need for blood transfusion is less in our present study.

There was a high success rate (94.7%) of B-Lynch suture in controlling atonic PPH, only one case needed a cesarean hysterectomy as bleeding persisted despite the application of B Lynch suture. There was one maternal mortality though atonic PPH was managed with B-Lynch suture patient but patient died in ICU due to heart disease. A study from Pakistan showed a success rate of 83% of B-Lynch suture in controlling PPH whereas another study showed a success rate of 91% in control of PPH.^18,19^ Although various studies showed B lynch was 100% effective in controlling atonic PPH,^20-22^ few others in the systematic review showed a 91.7% success rate in controlling PPH,^23-26^ thus most of them showing success rate between 82-95%.^12,26^

The difference in success rate may be due to different reasons, time of application, technique, patient selection criteria and disseminated intravascular coagulopathy features in patients. The B lynch brace suture has the advantage of being applied easily and rapidly. It should be attempted as early as possible in order to maximise its success, and prophylactic application should be considered in patients with high risk.^13^ Due to a higher success rate B Lynch suture can achieve remarkable results in the treatment of PPH, and can stop the bleeding quickly by its timely application. The post-graduate students, all trainees, and registrars in obstetrics and gynaecology should be taught the procedure so that it can be effectively used during emergencies.^11^

It is a single-centred study with small sample size. However, it highlights the prevalence of B Lynch suture and success in controlling atonic PPH. Despite the collection of various data regarding complications, it may not reflect the complication rate of the procedure. Thus larger population-based studies with long-term follow-up are recommended in future.

## CONCLUSIONS

The prevalence of the use of B-Lynch suture was similar to other studies done in similar settings. The B-Lynch suture is an easy method in controlling atonic primary PPH when medical management fails to control the haemorrhage and should always be considered before attempting a hysterectomy.
